# Renal Transcriptome Analysis of Programmed Hypertension Induced by Maternal Nutritional Insults

**DOI:** 10.3390/ijms160817826

**Published:** 2015-08-03

**Authors:** You-Lin Tain, Chien-Ning Hsu, Julie Y. H. Chan, Li-Tung Huang

**Affiliations:** 1 Departments of Pediatrics, Kaohsiung Chang Gung Memorial Hospital and Chang Gung University College of Medicine, Kaohsiung 833, Taiwan; E-Mail: litung.huang@gmail.com; 2 Center for Translational Research in Biomedical Sciences, Kaohsiung Chang Gung Memorial Hospital and Chang Gung University College of Medicine, Kaohsiung 833, Taiwan; E-Mail: jchan@adm.cgmh.org.tw; 3 Department of Pharmacy, Kaohsiung Chang Gung Memorial Hospital, Kaohsiung 833, Taiwan; E-Mail: Chien_ning_hsu@hotmail.com; 4 School of Pharmacy, Kaohsiung Medical University, Kaohsiung 807, Taiwan; 5 Department of Traditional Chinese Medicine, Chang Gung University, Linkow 244, Taiwan

**Keywords:** developmental programming, diabetes, fructose, hypertension, next generation sequencing, nutrition

## Abstract

Maternal nutrition can affect development, leading to long-term effects on the health of offspring. The most common outcome is programmed hypertension. We examined whether alterations in renal transcriptome are responsible for generating programmed hypertension among four different models using next-generation RNA sequencing (NGS) technology. Pregnant Sprague-Dawley rats received 50% caloric restriction (CR), intraperitoneal injection of 45 mg/kg streptozotocin, 60% high-fructose (HF) diet, or 1% NaCl in drinking water to conduct CR, diabetes, HF, or high-salt models, respectively. All four models induced programmed hypertension in adult male offspring. We observed 16 shared genes in a two-week-old kidney among four different models. The identified differential expressed genes (DEGs) that are related to the regulation of blood pressure included *Adrb3*, *Alb*, *Apoe*, *Calca*, *Kng1*, *Adm2*, *Guca2b*, *Hba2*, *Hba-a2*, and *Ppara*. The peroxisome proliferator-activated receptor (PPAR) signaling pathway and glutathione metabolism pathway were shared by the CR, diabetes, and HF models. Conclusively, a variety of maternal nutritional insults induced the same phenotype—programmed hypertension with differential alterations of renal transcriptome in adult male offspring. The roles of DEGs identified by the NGS in this study deserve further clarification to develop ideal maternal dietary interventions and thus spare the next generations from the burden of hypertension.

## 1. Introduction

Maternal nutrition plays an essential role in fetal growth, organogenesis, and development. Emerging evidence now supports that global burdens of non-communicable diseases may begin very early in life, and that exposure to unbalanced nutrition during prenatal and/or early postnatal development can predispose individuals to these diseases in the future lives of offspring [1,2]. One of the most common phenotypes of developmental programming is hypertension [3]. Nutritional epigenetics play a critical role during development and have been proposed to interpret the programming of hypertension [4]. Although several organs control blood pressure (BP), the kidney is particularly vulnerable to the insults of programming during nephrogenesis. Renal programming has been identified as a driving mechanism of programmed hypertension [5,6].

Recently we have shown that programmed hypertension developed in the male offspring of rats exposed to a variety of nutritional insults in pregnancy and lactation, including caloric restriction [7], maternal diabetes [8], and high fructose (HF) intake [9,10]. A number of mechanisms, including oxidative stress, a reduction in nephron numbers, alterations of the renin-angiotensin system (RAS) and sodium transporters, and epigenetic regulation in the kidney have been determined to interpret programmed hypertension [2,3,4,5,6]. However, each of the mechanisms determined in diverse models of developmental programming is unable entirely define the common genes and pathways that drive the programmed hypertension process.

Currently, only a few genome-wide studies have been conducted to capture the commonality in the renal transcriptome shared by various suboptimal conditions in pregnancy with similar phenotypes [11,12,13]. Since nephrogenesis occurs from late gestation to postnatal week 1–2 in rodents, analyzing renal transcriptome right after the completion of nephrogenesis to capture candidate genes and pathways might aid in identifying the primary programmed changes in response to different nutritional insults. Therefore, we employed the whole-genome next-generation RNA sequencing (NGS) to quantify the abundance of RNA transcripts in two-week-old offspring kidneys that had maternal exposure to caloric restriction, streptozotocin (STZ)-induced diabetes, HF, and high salt (HS). We hypothesize that there might be some gatekeeper genes/pathways in the kidney are common to all maternal nutritional insults. The aim of this research is to identify gatekeeper genes/pathways in the kidney, which is responsible for generating programmed hypertension among these four different models.

## 2. Results and Discussion

### 2.1. Hypertension Is a Common Phenotype in Response to Various Nutritional Insults

**Figure 1:**
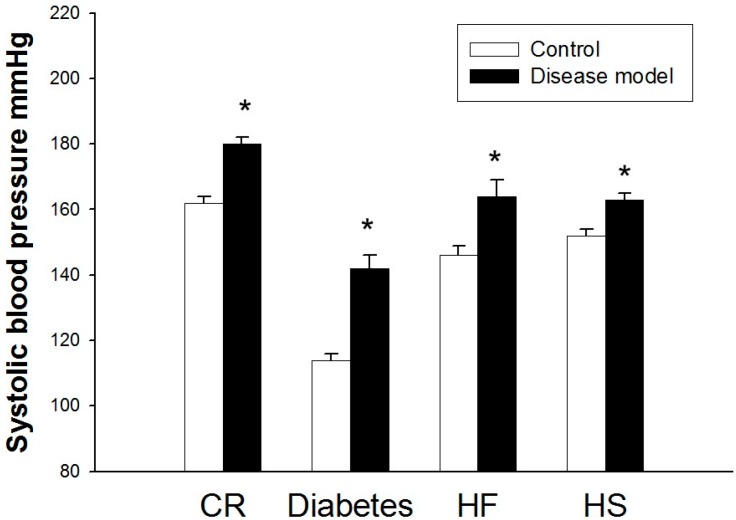
Effects of maternal caloric restriction (CR), streptozotocin-induced diabetes, high fructose (HF) intake, and high salt (HS) intake on systolic blood pressure in male adult offspring at 12 weeks of age. *n* = 6–10 per group. * *p* < 0.05 *vs.* respective control.

As shown in Figure 1, the systolic blood pressures (BP) of caloric restriction, maternal diabetes, HF, and HS groups were significantly greater than that of the control at 12 weeks of age. These data have been published previously and are included in this study for the sake of comparison [7,8,9,10]. The data described above indicate that a variety of nutritional insults in pregnancy induce the same phenotype in adult male offspring—programmed hypertension.

### 2.2. The Effects of Caloric Restriction (CR), Diabetes, High Fructose (HF), and High Salt (HS) on Renal Transcriptome

**Figure 2:**
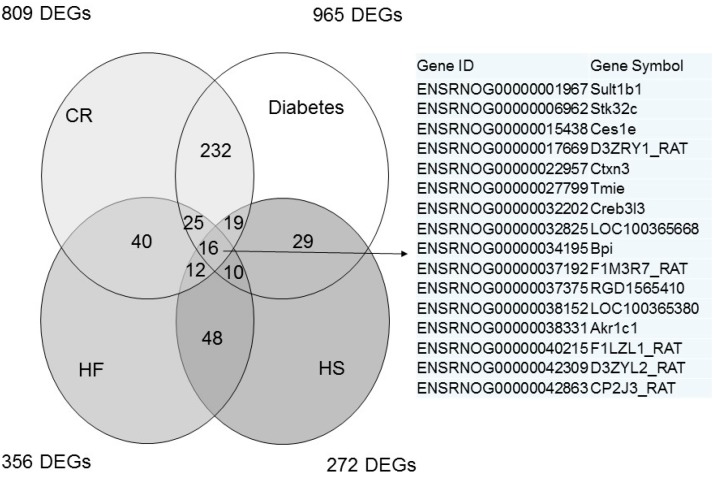
Venn diagram depicting unique and shared (overlapping circles) sets of differentially expressed genes (DEGs) in the kidney between CR, streptozotocin-induced diabetes, HF, and HS groups at two weeks of age. A total of 16 combined DEGs are listed in the right panel.

Next, we analyzed the differential gene expression induced by four different maternal insults in the offspring kidney. The mappability of genes in each group compared to the rat reference genome was 79.14% for the CR group, 78.08% for the diabetes group, 83.73% for the HF group, and 80.96% for the HS group, respectively. Among the differential expressed genes (DEGs), a total of 809 genes (CR *vs.* control) met the selection criteria of (1) genes that changed by reads per kilobase of transcript per million mapped reads (RPKM) >0.3 and (2) a minimum of two-fold difference in normalized read counts between groups. The *p* value was estimated for each gene and corrected for multiple testing by the Benjamini–Hochberg correction. The log2 fold change (FC) was used to partition the genes into up- and down-regulated groups. Next, 965, 356, and 272 DEGs were noted in the diabetes group *vs.* the control group, the HF group *vs.* the control group, and the HS group *vs.* the control group, respectively. Genes shared by different maternal insults are represented graphically by the Venn diagram shown in Figure 2.

Our results indicated that there are greater alterations of renal transcriptome in the CR and diabetes groups compared to the HF and HS groups at two weeks of age. A total of 16 DEGs were shared among four different models. To the best of our knowledge, none of them has shown a direct relationship with hypertension. We next used DAVID v6.7 to find functionally related gene groups and gain biological insight from our gene lists by identifying enriched Gene Ontology (GO) terms [14]. Among them, we found that 10 DEGs, namely *Adrb3*, *Alb*, *Apoe*, *Calca*, *Kng1*, *Adm2*, *Guca2b*, *Hba2*, *Hba-a2*, and *Ppara* were related to the regulation of BP (GO:0042311 vasodilation and GO:0045776 negative regulation of blood pressure).

Initially, we addressed whether there is a gatekeeper gene or pathway in the adult offspring kidney, which is responsible for generating programmed hypertension using three different models from maternal exposure to dexamethasone, HF, or *N*^G^-nitro-l-arginine-methyester (l-NAME, an inhibitor of nitric oxide synthase) [13]. Although we identified eight DEGs, including *Aqp2*, *Ptgs1*, *Eph2x*, *Hba-a2*, *Apln*, *Guca2b*, *Hmox1*, and *Npy*, that are related to the regulation of BP, it is possible that changes in transcriptome expression in adulthood might be a secondary phenomenon. We have now studied the alterations of renal transcriptome at two weeks of age, a period of time just after the completion of nephrogenesis, to identify primary programmed changes. It is noteworthy that *Hba-a2* and *Guca2b* are identified in both periods of time during development in different programming models. The *Guca2b* gene encodes the guanylate cyclase activator 2B. It is known for nitric oxide-mediated cyclic guanosine monophosphate production [15]. The *Hba-a2* gene encodes for hemoglobin α, adult chain 2, which is involved in the negative regulation of BP [16]. Because both genes are related to BP control, their roles in programmed hypertension deserve further investigation in other programmed models.

We also found 13 significantly related Kyoto Encyclopedia of Genes and Genomes (KEGG) pathways in the kidneys of CR and diabetes offspring at two weeks of age (Table 1). Next, there were eight and seven signaling pathways identified as the significant KEGG pathways in the kidneys of offspring exposed to HF and HS, respectively (Table 1). There was no KEGG pathway shared by four different models. However, the peroxisome proliferator-activated receptor (PPAR) signaling pathway and the glutathione metabolism pathway were shared by the CR, maternal diabetes, and HF models. It is interesting to note that the PPAR signaling pathway has been well studied in established hypertension and is a potential therapeutic target for hypertension [17,18]. From our NGS data, it seems reasonable to propose that PPAR signaling is a common pathway involved in the development of hypertension. Next, oxidative stress has been proposed as an important link between renal programming and hypertension [6]. Glutathione (GSH) is the major intracellular antioxidant [19]. Our previous study demonstrated that *N*-acetylcysteine can increase GSH and reduce oxidative stress to prevent the development of hypertension in young spontaneously hypertensive rats [20]. Therefore, additional studies are required to unravel the underlying mechanisms of the glutathione pathway related to programmed hypertension.

**Table 1:** Significantly regulated Kyoto Encyclopedia of Genes and Genomes (KEGG) pathways in the two-week-old offspring kidneys of maternal CR, diabetes, HF, and HS.

Term	Count	%	*p*-Value	Benjamini	
CR					
Ribosome	22	3.0	1.1 × 10^−14^	1.4 × 10^−12^	
Cell cycle	16	2.2	3.6 × 10^−6^	2.4 × 10^−4^	
Oocyte meiosis	9	1.2	1.6 × 10^−2^	5.3 × 10^−1^	
DNA replication	5	0.7	1.9 × 10^−2^	4.8 × 10^−1^	
Fatty acid metabolism	5	0.7	3.4 × 10^−2^	6.1 × 10^−1^	
Tryptophan metabolism	5	0.7	3.7 × 10^−2^	5.7 × 10^−1^	
Homologous recombination	4	0.5	4.0 × 10^−2^	5.5 × 10^−1^	
Progesterone-mediated oocyte maturation	7	0.9	4.3 × 10^−2^	5.2 × 10^−1^	
Valine, leucine, and isoleucine degradation	5	0.7	4.6 × 10^−2^	5.1 × 10^−1^	
Prostate cancer	7	0.9	4.9 × 10^−2^	5.0 × 10^−1^	
PPAR signaling pathway	6	0.8	5.8 × 10^−2^	5.2 × 10^−1^	
Glutathione metabolism	5	0.7	5.9 × 10^−2^	5.0 × 10^−1^	
Arginine and proline metabolism	5	0.7	7.0 × 10^−2^	5.3 × 10^−1^	
Diabetes					
Ribosome	14	1.6	2.6 × 10^−6^	3.8 × 10^−4^	
ABC transporters	7	0.8	3.0 × 10^−3^	2.0 × 10^−1^	
Complement and coagulation cascades	8	0.9	8.0 × 10^−3^	3.3 × 10^−1^	
Spliceosome	10	1.1	1.8 × 10^−2^	4.9 × 10^−1^	
Antigen processing and presentation	8	0.9	2.6 × 10^−2^	5.4 × 10^−1^	
Prostate cancer	8	0.9	2.9 × 10^−2^	5.1 × 10^−1^	
Drug metabolism	7	0.8	3.1 × 10^−2^	4.9 × 10^−1^	
Histidine metabolism	4	0.4	4.3 × 10^−2^	5.6 × 10^−1^	
Metabolism of xenobiotics by cytochrome P450	6	0.7	4.7 × 10^−2^	5.5 × 10^−1^	
ECM-receptor interaction	7	0.8	5.1 × 10^−2^	5.4 × 10^−1^	
Tryptophan metabolism	5	0.6	5.3 × 10^−2^	5.1 × 10^−1^	
Glutathione metabolism	5	0.6	8.2 × 10^−2^	6.5 × 10^−1^	
PPAR signaling pathway	6	0.7	8.5 × 10^−2^	6.3 × 10^−1^	
HF				
PPAR signaling pathway	9	2.7	9.2 × 10^−6^	8.7 × 10^−4^
Butanoate metabolism	4	1.2	1.3 × 10^−2^	4.6 × 10^−1^
Arachidonic acid metabolism	5	1.5	2.1 × 10^−2^	4.8 × 10^−1^
Fatty acid metabolism	4	1.2	2.5 × 10^−2^	4.5 × 10^−1^
Glutathione metabolism	4	1.2	3.9 × 10^−2^	5.3 × 10^−1^
Metabolism of xenobiotics by cytochrome P450	4	1.2	6.1 × 10^−2^	6.3 × 10^−1^
Tyrosine metabolism	3	0.9	9.2 × 10^−2^	7.3 × 10^−1^
Drug metabolism	4	1.2	9.3 × 10^−2^	6.9 × 10^−1^
HS				
Cell adhesion molecules (CAMs)	8	3.3	3.0 × 10^−4^	2.3 × 10^−2^
Complement and coagulation cascades	4	1.6	2.4 × 10^−2^	6.1 × 10^−1^
Hematopoietic cell lineage	4	1.6	3.2 × 10^−2^	5.6 × 10^−1^
Systemic lupus erythematosus	4	1.6	4.6 × 10^−2^	5.9 × 10^−1^
Intestinal immune network for IgA production	3	1.2	6.1 × 10^−2^	6.1 × 10^−1^
Graft-*versus*-host disease	3	1.2	7.6 × 10^−2^	6.3 × 10^−1^
Allograft rejection	3	1.2	8.1 × 10^−2^	6.0 × 10^−1^

### 2.3. The Effects of CR, Diabetes, HF, and HS on Nephrogenesis-Related Gene Expression

Our previous reports showed that CR induced a low nephron number in male offspring at 12 weeks of age and seven-day-old STZ offspring also had low nephron numbers [8,21]. Given that renal programming may manifest as a low nephron endowment in nutrition-induced programming models [5,6], and that low nephron numbers play a role in programmed hypertension [5,6], we thus analyzed a panel of genes that has previously been reported to be relevant to kidney development in offspring kidneys in this study [9,22,23].

As shown in Table 2, *Ret*, *Gdnf*, *Gata3*, *Lif*, *Fgf2*, and *Osr1* were found modified above the chosen threshold in the kidneys of offspring at two weeks of age in response to maternal CR. In addition, *Fgf10*, *Gdnf*, *Spry3*, *Six1*, *Grem1*, *Wnt11*, *Fgf2*, *Etv4*, *Osr1*, *Bmp2*, *Igf1*, and *Notch3* had significant differential expression in response to maternal diabetes. On the other hand, the observed transcription alterations were mild in the HF and HS models. These findings are consistent with our previous reports showing that low nephron numbers developed in the male offspring exposed to maternal CR and diabetes, but not HF and HS. Interestingly, *Gdnf* was commonly shared by the four different models. The *Gdnf* gene encodes for glial cell line-derived neurotrophic factor (GDNF), which is required for the morphogenesis of the ureteric bud during kidney development [24]. Given that a low nephron number was found in GDNF^+/−^ heterozygous mice [25], and that hypoxic conditions increased *Gdnf* expression and ureteric bud branching, our recent data suggest that increased *Gdnf* expression is a compensatory mechanism in response to impaired nephrogenesis in differently programmed models. Next, we observed that *Fgf2* and *Osr1* were shared by CR and maternal diabetes, while *Six1* was shared by diabetes and HF. Whether these genes are commonly related to low nephron numbers in response to different maternal insults awaits further evaluation.

Our NGS data indicate that a diverse range of maternal nutritional insults might generate differentially programmed processes despite a similar phenotype. As we analyzed transcriptome right after the completion of nephrogenesis, the observed alteration of renal transcriptome is likely a consequence of renal development, and a cause of programmed hypertension. However, we cannot absolutely rule out the possibility that transcriptome changes occurring early during nephrogenesis will persist until later in life. Because transcriptome expression in later life could be the consequence of developmental programming, analyzing transcriptome at different developmental windows might aid in identifying persistently programmed changes in response to early insults. Another limitation in this study is that we did not examine alterations of renal transcriptome in different windows of exposure to each insult. Given that epigenetic regulation varies during different developmental windows, whether the same nutritional insult leads to differentially regulated genes between diverse windows of exposure is worthy of further study.

**Table 2:** Fold changes in genes involved in kidney development in the kidneys of offspring at two weeks of age exposed to maternal CR, diabetes, HF, and HS.

Gene ID	Gene Symbol	CR	Diabetes		HF		HS	
Expansion and survival of renal stem cells								
ENSRNOG00000009425	*Fgf7*	0.61	0.57		1.31		1.34	
ENSRNOG00000012278	*Fgf10*	0.52	**0.38**		0.55		0.87	
ENSRNOG00000016050	*Fgfr1*	1.08	1.33		0.87		1.03	
ENSRNOG00000016374	*Fgfr2*	0.72	1.28		0.89		0.99	
ENSRNOG00000009972	*Rara*	0.95	1.38		1.04		1.28	
Formation and extension of the primary nephric duct								
ENSRNOG00000013074	*Wt1*	1.16	1.51		0.98		1.13	
ENSRNOG00000014751	*Ret*	3.3	1.06		1.56		1.75	
ENSRNOG00000012819	*Gdnf*	**2563**	**1508**		**1836**		**1362**	
ENSRNOG00000017438	*Gfra1*	1.18	1.28		1.64		1.38	
ENSRNOG00000003840	*Slit2*	1.05	1.17		0.75		0.86	
ENSRNOG00000029598	*Robo2*	0.99	1.45		0.85		0.79	
ENSRNOG00000009694	*Bmp4*	0.81	0.93		0.88		1.01	
ENSRNOG00000025371	*Spry1*	1.14	0.77		0.85		1.32	
ENSRNOG00000010058	*Spry2*	1.4	1.26		1.02		1.17	
ENSRNOG00000008430	*Spry3*	ND	**123**		1.04		278	
ENSRNOG00000013851	*Spry4*	1.23	0.86		1.21		1.41	
ENSRNOG00000022777	*Six1*	1.58	**0.4**		**2.64**		1.55	
ENSRNOG00000007590	*Eya1*	0.62	0.79		1.3		1.28	
ENSRNOG00000019336	*Gata3*	0.37	0.58		0.71		0.76	
ENSRNOG00000026053	*Grem1*	0.57	**0.47**		1.21		0.83	
Glomerular development								
ENSRNOG00000019598	*Vegfa*	0.87	0.88		1.22		1.24	
ENSRNOG00000016700	*Pod1/Tcf21*	1.14	1.1		0.81		0.91	
ENSRNOG00000001312	*Pdgfa*	1.15	1.5		1		1.09	
ENSRNOG00000017197	*Pdgfb*	0.93	1.28		0.9		1.24	
ENSRNOG00000011987	*Cd2ap*	0.69	0.67		0.95		0.8	
ENSRNOG00000014917	*Nck1*	0.81	0.61	0.98		0.83	
ENSRNOG00000017155	*Nck2*	1.13	1.31	1.1		1.29	
ENSRNOG00000016408	*Kirrel*	1.18	1.67	0.82		1.18	
ENSRNOG00000016281	*Col4a1*	0.94	1.38	0.88		0.99	
ENSRNOG00000023972	*Col4a2*	1.09	1.75	0.77		0.91	
ENSRNOG00000014851	*Col4a4*	1.14	1.07	0.8		1	
ENSRNOG00000018951	*Col4a5*	1.36	1.52	0.85		0.96	
Initiation of metanephric development							
ENSRNOG00000020417	*Gsk3a*	0.93	0.9	1.08		1.22	
ENSRNOG00000002833	*Gsk3b*	0.73	1.09	1		1.07	
ENSRNOG00000013166	*Wnt4*	1.1	1.9	0.7		1.12	
ENSRNOG00000003807	*Wnt9b*	0.75	1.27	**0.49**		0.85	
ENSRNOG00000015982	*Wnt11*	1.18	**3.37**	1.29		1.3	
ENSRNOG00000007002	*Lif*	**0.36**	0.64	0.95		1.12	
ENSRNOG00000017392	*Fgf2*	**2.07**	**2.9**	1.54		0.82	
ENSRNOG00000020652	*Tgfb1*	0.73	0.81	0.88		1.14	
ENSRNOG00000003866	*Cxcr4*	1.12	1.06	0.95		0.72	
ENSRNOG00000020792	*Etv4*	0.94	**2.66**	1.7		1.53	
ENSRNOG00000001785	*Etv5*	0.98	1.46	1.05		1.23	
ENSRNOG00000016848	*Fzd4*	1.08	1.05	0.93		0.94	
ENSRNOG00000038571	*Fzd8*	0.87	1.08	0.79		0.93	
Mesoderm patterning							
ENSRNOG00000004210	*Osr1*	**0.27**	**0.46**	0.61		0.57	
ENSRNOG00000014253	*Pax2*	0.7	1.02	0.89		1.24	
ENSRNOG00000026203	*Pax8*	0.75	1.04	0.91		1.16	
ENSRNOG00000002812	*Lhx1*	0.99	1.35	0.99		1	
ENSRNOG00000021276	*Bmp2*	1.72	**2.39**	0.86		1.05	
ENSRNOG00000000109	*Fgf20*	ND	ND	ND		ND	
ENSRNOG00000006530	*Hoxa6*	0.97	1.32	0.96		0.98	
ENSRNOG00000000556	*Nodal*	ND	ND	ND		**242**	
ENSRNOG00000017800	*Foxc1*	1.45	1.49	1.01		1.21	
ENSRNOG00000009482	*Emx2*	0.77	0.77	1		1.01	
Nephron development							
ENSRNOG00000004517	*Igf1*	0.55	**0.44**	0.77		0.64	
ENSRNOG00000019322	*Notch1*	0.81	1.14	0.89		1.04	
ENSRNOG00000018835	*Notch2*	1.17	1.58	0.74		0.98	
ENSRNOG00000004346	*Notch3*	1.18	**2.09**	0.83		1.06	
ENSRNOG00000000442	*Notch4*	1.32	1.11	0.9		1.07	
ENSRNOG00000020155	*Jnk1/Mapk8*	0.87	0.91	0.85		0.8	
ENSRNOG00000002079	*Jnk3/Mapk10*	0.51	0.53	0.79		0.91	
ENSRNOG00000013535	*Cdh6*	0.66	0.87	0.77		0.83	
ENSRNOG00000013481	*Cdh11*	0.9	1.38	0.74		0.85	

ND = not detectable; Significant results are highlighted in bold.

## 3. Experimental Section

### 3.1. Animals and Experimental Design

This study was approved and performed under the Guidelines for the Care and Use of Laboratory Animals of the National Institutes of Health and the Institutional Animal Care and Use Committee of the Kaohsiung Chang Gung Memorial Hospital (Permit Numbers: 2008070201, 2012101601 and 2011120602). Sprague Dawley (SD) rats (10 weeks old) were obtained (BioLASCO Taiwan Co., Ltd., Taipei, Taiwan), and then housed and maintained in a facility accredited by the Association for Assessment and Accreditation of Laboratory Animal Care International. Male rats were caged with individual females until mating was confirmed.

In the CR model, food-restricted maternal SD rats received 11 g/d of a standard chow from day 11 of pregnancy until the day of delivery (day 23) and 20 g/d of the same chow during the entire lactation period. This scheme ensures that the rats receive 50% of the ad libitum food intake as we reported previously [7]. The control group consisted of the male offspring of mother rats with free access to standard rat chow. For the diabetic model, pregnant SD rats were made diabetic on day 0 of gestation by a single intraperitoneal injection of 45 mg STZ (freshly dissolved in citrate buffer) per kilogram of body weight as we reported previously [8]. Control rats were given an equivalent amount of citrate buffer. In the HF model, pregnant SD rats received regular chow or chow supplemented with fructose (60% diet by weight) during the whole period of pregnancy and lactation [9]. To conduct the HS model, pregnant rats received NaCl (1%) in drinking water during the whole period of pregnancy and lactation [10]. BP was measured in conscious rats by an indirect tail-cuff method (BP-2000, Visitech Systems, Inc., Apex, NC, USA) [7,8,9,10]. To ensure accuracy and reproducibility, 1 week prior to the experiment the rats were trained to restraint and tail-cuff inflation. BP measurements were taken between 1300 and 1700 each day. Rats were placed on specimen platform. Their tails were passed through tail cuffs and secured in place with tape. Following a 10-min warm-up period, 10 preliminary cycles of tail-cuff inflation were performed. For each rat, 5 cycles were recorded at each time point. Average of values from three stable measurements were taken. Three male offspring in each group were sacrificed at 2 weeks of age. The others were sacrificed at 12 weeks of age. Male offspring were euthanized by an i.p. overdose of pentobarbital. The midline of the abdomen was opened, and the intestines were displaced laterally to allow visualization of the aorta. Later the aorta was dissected and cannulated with a 20- to 23-gauge butterfly, heparinized blood samples were collected, the vena cava was cut, and PBS was perfused until the kidneys were blanched. Perfused kidneys were harvested, decapsulated, divided into cortex and medulla, flash frozen in liquid nitrogen, and stored at −80 °C freezer for further analysis.

### 3.2. Next-Generation Sequencing and Analysis

To minimize the variation among individual rats, kidney cortex samples from three rats per group were pooled. Total RNA from pooled kidney samples were used for whole-genome RNA next-generation sequencing (RNA-Seq), performed by Welgene Biotech Co., Ltd. (Taipei, Taiwan). Purified RNA was quantified at 260 nm (OD_600_) by using a ND-1000 spectrophotometer (Nanodrop Technology, Wilmington, DE, USA) and analyzed using a Bioanalyzer 2100 (Agilent Technology, Santa Clara, CA, USA) with RNA 6000 LabChip kit (Agilent Technologies, Santa Clara, CA, USA) as we previously described [13]. All procedures were performed according to the Illumina protocol. For all samples, library construction was performed using the TruSeq RNA Sample Prep Kit v2 for ~160 bp (single-end) sequencing and the Solexa platform. The sequence was directly determined by sequencing-by-synthesis technology using the TruSeq SBS Kit (Illumina Inc., San Diego, CA, USA). Raw sequences were obtained using the Illumina GA Pipeline software CASAVA v1.8 (Illumina Inc., San Diego, CA, USA), which was expected to generate 30 million reads per sample. Quantification for gene expression was calculated as reads per kilobase of exon per million mapped reads (RPKM). Cufflink v 2.1.1 and CummeRbund v 2.0.0 (Illumina Inc., San Diego, CA, USA) were used to perform statistical analyses of the gene expression profiles. The output files were further annotated by adding gene functional descriptions and GO classifications. The reference genome and gene annotations were retrieved from the Ensembl database [26]. GO term enrichment and fold enrichment or depletion for gene lists of significantly up- and down-regulated genes in kidneys were determined. GO analysis for significant genes was performed using KEGG [27] and NIH DAVID Bioinformatics Resources 6.7 to identify functionally related gene groups [14].

### 3.3. Statistical Analysis

Data were represented as mean ± S.E.M. BP was analyzed by using the Student *t*-test to compare the disease model group with the respective control group. A *p*-value <0.05 was considered statistically significant. All analyses were performed using the Statistical Package for the Social Sciences software (SPSS, IBM, Armonk, NY, USA).

## 4. Conclusions

In conclusion, a variety of maternal nutritional insults induce the same phenotype—programmed hypertension in adult male offspring. Using NGS technology, we identified 16 genes shared by 4 different models. In addition, 10 DEGs, namely *Adrb3*, *Alb*, *Apoe*, *Calca*, *Kng1*, *Adm2*, *Guca2b*, *Hba2*, *Hba-a2*, and *Ppara*, were related to the regulation of BP. Moreover, the PPAR signaling pathway and the glutathione metabolism pathway were shared by the CR, maternal diabetes, and HF models. Our NGS results are of significance to the development of maternal dietary interventions in the prevention of renal programming to reduce the global burden of hypertension.
